# Parliament and the Profession

**Published:** 1921-05-14

**Authors:** 


### Parliament and the Profession.

### Dentists Bill.

The Minister of Health announced that a Dentists Bill will be introduced at an early date during the present session, and that, if there is no opposition to the second reading, it may be found possible to proceed with it.

### Committee on Voluntary Hospitals.

Sir Alfred Mond stated that it is expected that the Committee on Voluntary Hospitals will present their report by the end of May.

